# Gastric necrosis following a hiatal hernia: A case report

**DOI:** 10.1016/j.ijscr.2020.12.092

**Published:** 2021-01-06

**Authors:** Gregoire Longchamp, Axel Andres, Ziad Abbassi

**Affiliations:** Division of Digestive Surgery, University Hospitals of Geneva, 1205, Geneva, Switzerland

**Keywords:** SCARE, surgical case report, CT, computed tomography, Hiatal hernia, Gastric volvulus, Gastric necrosis, Fundoplication, Case report

## Abstract

**Introduction:**

Gastric volvulus are rare. Complications can be life threatening, including necrosis and perforation. Assessment of mucosal viability is essential, and urgent surgical intervention is mandatory in case of vascular compromise.

**Presentation of case:**

An 72-year-old female known for a paraesophageal hiatal hernia was admitted at our emergency department with acute abdominal pain. Blood count demonstrated leukocytosis and increased C-reactive protein. Abdominal computed tomography showed a mesenteroaxial gastric volvulus. Urgent upper endoscopy revealed mucosal ischemia, which prompted immediate laparotomy with partial gastrectomy, cruroplasty, and Dor fundoplication. Postoperative course was uneventful.

**Discussion:**

Gastric volvulus is initially treated with nasogastric tube decompression, but definitive treatment is achieved surgically. When there is an associated hernia, closing the anatomical defect and fundoplication should be performed. Complication such as necrosis is associated with a high mortality, and requires urgent surgical repair.

**Conclusion:**

Gastric volvulus can be life-threatening. Urgent endoscopic or surgical assessment should be conducted to assess mucosal viability.

## Highlights

• Nasogastric tube decompression if the first-step treatment of gastric volvulus, which can be placed blindly or under endoscopic guidance.
• Prompt endoscopic or surgical assessment should be conducted when signs of sepsis, perforation, or ischemia are present.
• Definitive treatment for secondary volvulus is best achieved with hernia reduction, closing of the anatomical defect, and fundoplication.
• Primary volvulus can be treated with gastropexy alone.

## 1 Introduction

Gastric volvulus is a rare condition where the stomach twists on itself. Volvulus can be partial, manifesting with mild symptoms; or complete, presenting as an acute obstruction. It can lead to severe complications, such as gastric necrosis and perforation [1]. Physicians should be aware of this condition, and undertake prompt and appropriate management. Herein, we report a case of a mesenteroaxial volvulus leading to gastric necrosis, using the Surgical Case Report (SCARE) guidelines (Supplementary table 1) [2].

## 2 Presentation of case

**Fig. 1:**
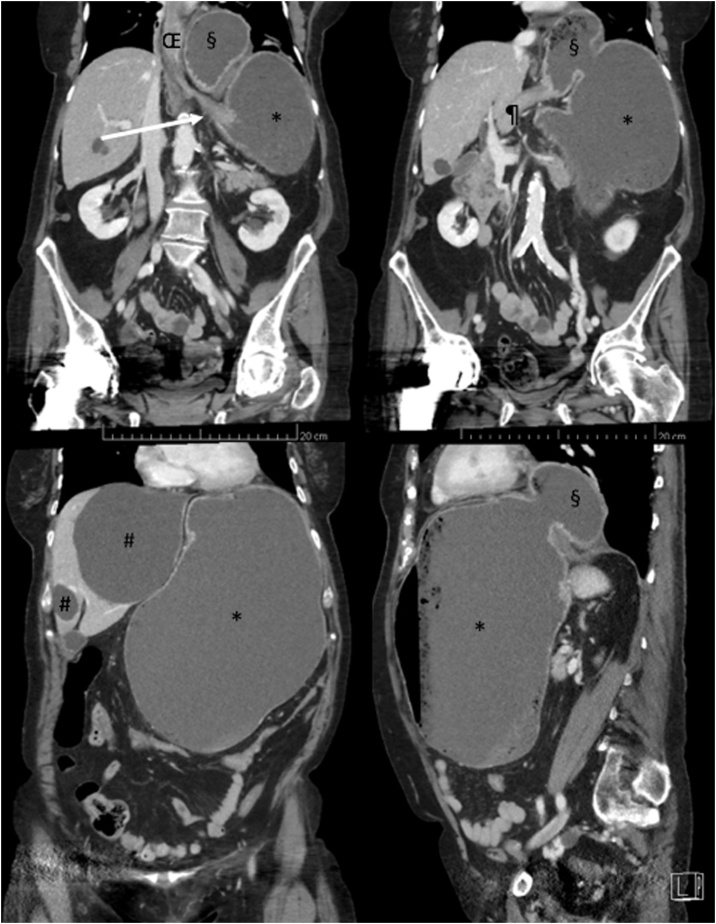
Abdominal CT (coronal view on the upper left, upper right, lower left; and sagittal view on the lower right) at initial presentation showing the mesenteroaxial gastric volvulus with the antrum (§) above the gastroesophageal junction (white arrow), exerting a mass effect on the distal esophagus (Œ). Other symbols refer to duodenum (¶), biliary cysts (#) and distended stomach (*).

An 72-year-old women patient presented to the emergency department of a tertiary hospital, with acute abdominal pain, nausea, and vomiting. Her past medical history included a medically treated paraesophageal hiatal hernia for four years, biliary cysts, and appendicectomy during childhood. On admission, vital signs were: pulse rate of 120 beats per min, blood pressure of 133/85 mmHg, respiratory rate of 18 breaths per minute, temperature of 37.0 °C, and oxygen saturation of 97% on ambiant air. Physical examination revealed abdominal distension with reduced bowel sounds, diffuse abdominal pain without rebound tenderness or guarding. Laboratory studies showed leukocytosis (23 G/l), mild elevated C-reactive protein (28 mg/L), normal lactate (1.1 mmol/l). Computed tomography (CT) revealed a mesenteroaxial gastric volvulus associated with a paraesophageal hernia (Fig. 1), and a significant distended stomach, without sign for perforation nor ischemia (i.e. no pneumatosis, no free air or fluid, no lack of contrast enhancement of the gastric wall). Biliary cysts were stable in size from previous imaging studies.

**Fig. 2:**
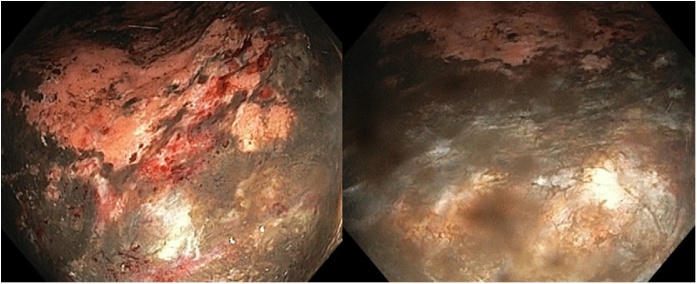
Upper endoscopy showing evidence of mucosal ischemia, with visualization of the underlying muscular layer.

**Fig. 3:**
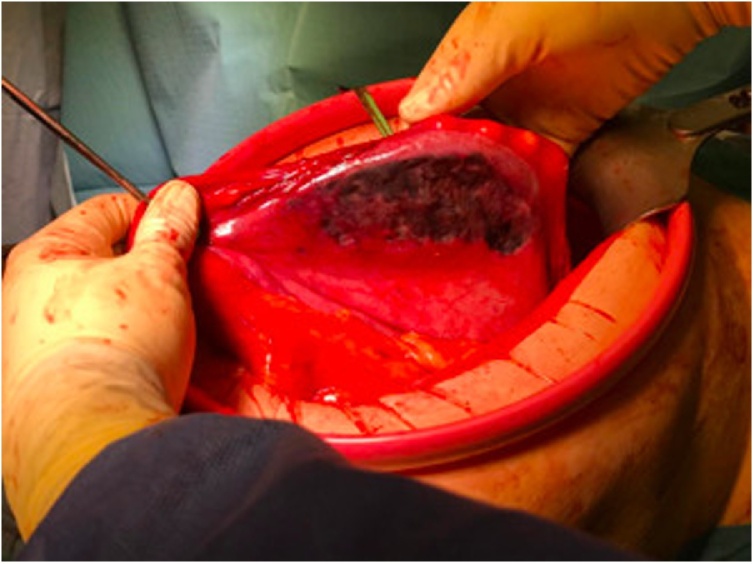
Gastric necrosis found during laparotomy.

After an unsuccessful blind attempt, the patient underwent emergency gastroscopy for nasogastric tube placement under endoscopic guidance, and to allow assessment of gastric mucosa viability. The latter showed gastric mucosa ischemia in the proximal third of the paraesophageal hernia, with appearance of the underlying muscular layer (Fig. 2). Directly after gastroscopy, the patient underwent an exploratory laparotomy, confirming necrosis of the stomach (Fig. 3). The paraesophageal hernia was reduced and the enlarged hiatus was closed with a suture cruroplasty. An indocyanine green test was conducted to assess the extent of the devascularization, with consecutive segmental gastrectomy, followed by a Dor fundoplication. The procedure was performed by a board-certified gastrointestinal surgeon.

**Fig. 4:**
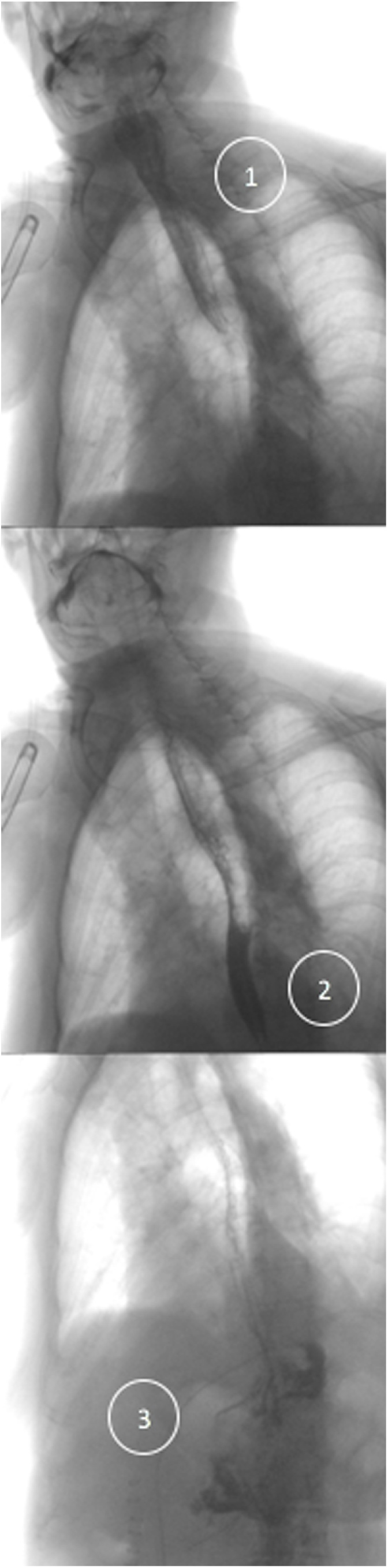
Gastrografin swallow study showing no complication: 1: gastrografin in the proximal esophagus, 2: gastrografin in the distal esophagus, 3: gastrografin in the stomach.

Postoperative course on the surgical ward was uneventful. Gastrografin swallow study on postoperative day three showed no sign of complications (Fig. 4), and the patient was discharge on postoperative day six. Outpatient followed-up at one month revealed no complain and well-tolerated feeding.

## 3 Discussion

Gastric volvulus is a rare condition, mainly reported from case reports and series. A literature review performed between 1999 and 2018 identified 43 cases of gastric volvulus [3]. Many risk factors were reported, including age > 50 years and hiatal hernia, which were found in the present case. Gastric volvulus can be classified as follows [1,3]:

1. *Primary volvulus (or idiopathic)* caused by deficiency of the stomach fixation, i.e. ligamentous laxity. *Secondary volvulus* following anatomical abnormality of surrounding structures, i.e. paraesophageal hiatal hernia or diaphragmatic hernia;
2. *Mesenteroaxial volvulus (type I)* with rotation around the short axis of the stomach, placing the antrum above the gastroesophageal junction. *Organoaxial volvulus (type II)* with rotation along the long axis, the greater curvature being displaced above the lesser curvature, also known as upside-down stomach. *Mixed volvulus (type III)* when both components are present, and *unclassified volvulus (type IV)*.

Manifestations of gastric volvulus include pain in the lower abdomen of lower chest, nausea, vomiting, and hematemesis. In cases of incomplete volvulus, symptoms may be chronic or mild, and a history of anatomical anomaly helps for the diagnosis. Two thirds of patients will have the Borchardt’s triad consisting of pain, vomiting, and inability to pass a nasogastric tube [4]. Vascular compromise may result in mucosal ischemia, necrosis, and perforation which may manifest with signs of peritonitis on abdominal examination, abnormal vital signs, or increased blood lactate. CT-scan is valuable in the diagnosis of volvulus, and can identify ischemia, or gastric wall perforation. However, signs of complications may be absent, and prompt endoscopic or surgical assessment should not be delayed, as demonstrated in our case.

Nasogastric tube decompression if the first-step treatment of gastric volvulus, which can be placed blindly or under endoscopic guidance. In case of symptomatic resolution after decompression, without signs for ischemia or perforation, elective surgical repair can be considered. However, endoscopic or surgical assessment should not be delayed in case of sepsis, perforation, or ischemia, associated with a 30% mortality rate [5]. Definitive treatment for secondary volvulus is best achieved with hernia reduction, closing of the anatomical defect, and fundoplication. In case of primary volvulus, gastropexy alone may be sufficient. In a cohort of 30 patients with intrathoracic stomach including 14 gastric volvulus, laparoscopic treatment showed to be effective and safe. In the latter study, only one case recurred, with a 30-day complication rate of 11%, and no death at a mean follow-up of 11 months [6]. Thus, the choice between open and laparoscopic approach relies on the patient previous intervention, the presence of hemodynamic instability, abdominal contamination, and surgeon experience. For patients with surgical contraindication, percutaneous gastrostomy is an alternative.

## 4 Conclusion

Gastric volvulus is a life-threatening condition, that can be initially treated with decompression. Urgent endoscopic or surgical assessment should be conducted early to assessed mucosa viability.

## Declaration of Competing Interest

None.

## Funding

None.

## Ethical approval

The study is exempt from ethical approval.

## Consent

Written informed consent was obtained from the patient for publication of this case report and accompanying images. A copy of the written consent is available for review by the Editor-in-Chief of this journal on request.

## Author contribution

GL and ZA conceived and designed the study.

GL and ZA acquired the data.

GL, AA, ZA interpreted the data.

GL, AA, ZA contributed to the writing of the manuscript and to its critical revision.

GL, AA, ZA approved the final version of the manuscript.

## Registration of research studies

1. Name of the registry: not applicable.
2. Unique identifying number or registration ID: not applicable.
3. Hyperlink to your specific registration (must be publicly accessible and will be checked): not applicable.

## Guarantor

Gregoire Longchamp.

## Provenance and peer review

Not commissioned, externally peer-reviewed.
